# Correlation between Hepatocyte Growth Factor (HGF) with D-Dimer and Interleukin-6 as Prognostic Markers of Coagulation and Inflammation in Long COVID-19 Survivors

**DOI:** 10.3390/cimb45070361

**Published:** 2023-07-08

**Authors:** Bena Zaira, Trilis Yulianti, Jutti Levita

**Affiliations:** 1 Student at Master Program in Clinical Pharmacy, Faculty of Pharmacy, Universitas Padjadjaran, Sumedang 45363, Indonesia; bena20001@mail.unpad.ac.id; 2 Prodia Education and Research Institute, Jakarta 10430, Indonesia; trilisyulianti@gmail.com; 3 Department of Pharmacology and Clinical Pharmacy, Padjadjaran University, Sumedang 45363, Indonesia

**Keywords:** blood coagulation, COVID-19, D-dimer, hepatocyte growth factor, interleukins, pro-inflammatory cytokines, SARS-CoV-2

## Abstract

In general, an individual who experiences the symptoms of Severe Acute Respiratory Syndrome Coronavirus 2 or SARS-CoV-2 infection is declared as recovered after 2 weeks. However, approximately 10–20% of these survivors have been reported to encounter long-term health problems, defined as ‘long COVID-19’, e.g., blood coagulation which leads to stroke with an estimated incidence of 3%, and pulmonary embolism with 5% incidence. At the time of infection, the immune response produces pro-inflammatory cytokines that stimulate stromal cells to produce pro-hepatocyte growth factor (pro-HGF) and eventually is activated into hepatocyte growth factor (HGF), which helps the coagulation process in endothelial and epithelial cells. HGF is a marker that appears as an inflammatory response that leads to coagulation. Currently, there is no information on the effect of SARS-CoV-2 infection on serum HGF concentrations as a marker of the prognosis of coagulation in long COVID-19 survivors. This review discusses the pathophysiology between COVID-19 and HGF, IL-6, and D-dimer.

## 1. Introduction

The outbreak of COVID-19 due to SARS-CoV-2 infection, which first appeared in Wuhan, China, in December 2019, had strongly damaged the health sector. Globally, over 2.8 million new cases and almost eighteen thousand deaths were reported from 20 March to 16 April 2023. Contradictory to the global trend, a significant elevation in cases and deaths was reported to occur in Southeast Asian and Eastern Mediterranean areas [1,2]. At present, WHO is tracing two variants of interest, namely XBB.1.5 and XBB.1.16. XBB.1.5 has spread in 96 countries, whereas XBB.1.16 is observed in 31 countries [2].

The SARS-CoV-2 spike or S protein (S1) binds to the many ACE2 receptors on the respiratory epithelium, including type II alveolar epithelial cells, to allow SARS-CoV-2 access into the hosts’ cells. In addition to the respiratory epithelium, the upper esophagus, enterocytes from the ileum, cells in the cardiac, proximal tubular cells in the kidney, and urothelial cells of the bladder all express ACE2 receptors. The host transmembrane serine protease 2 (TMPRSS2) primes the spike protein S2 subunit after the viral attachment process, which enables cell entrance and subsequent viral replication endocytosis with the formation of virions [3]. When the viral RNA enters the host, polyprotein 1a/1ab (pp1a/pp1ab) is created and starts the replication. The subgenomic RNA (sgRNA) sequences are produced during transcription through the replication–transcription complex (RCT), which is arranged in double-membrane vesicles. Contrarily, transcription is terminated at transcription regulatory sequences, which are positioned in between the so-called open reading frames (ORFs) that serve as templates for the synthesis of subgenomic mRNAs. There can be at least six ORFs in an abnormal CoV genome. Other ORFs, in addition to ORF1a and ORF1b, encode structural proteins such as spike, membrane, envelope, and nucleocapsid proteins, as well as auxiliary protein chains. Unique structural and auxiliary proteins are translated by specialized sgRNAs in various CoVs. The NSP and structural proteins’ roles in CoV and SARS-CoV-2 pathogenesis are connected [4]. The spike RBD permits binding to the ACE2 receptor in the tissues, including the lungs. The polybasic site of an amino acids spike protein enables the human enzyme furin to metabolize the amino acid in a useful manner (protease). This procedure makes it possible for the fusing of the viral and cell membranes, which is required for the virus to pass through the cell and expose the fusion sequences [5].

However, about 80% of individuals with COVID-19 had reported mild to moderate symptoms, and 5% underwent severe illness [6]. Then, up to 70% of COVID-19 survivors may develop long-term medical consequences in addition to significant morbidity and mortality in the initial few weeks following infection. Long after patients are virus-free, the persistent symptoms from COVID-19 infection can drastically lower the quality of life. The long-term COVID-19 effect or extended COVID-19 is another name for this post-COVID-19 situation [7]. This condition is known as “long COVID”, “long haulers”, or “post-COVID syndrome” [8,9,10]. In May 2020, the phrase “long COVID” was first used [11]. 

There are a lot of COVID-19 patients who improve their health several weeks after contracting the virus, although a few develop severe post-COVID-19 disorders. Patients may develop post-COVID-19 conditions four weeks or more following their first SARS-CoV-2 infection. These conditions can include a variety of new health problems, returning health problems, recurrences, or persistent health problems. Within days or weeks of healing, even those who do not exhibit COVID-19 symptoms may develop a post-COVID-19 disease [12]. 

Currently, there is no information on the effect of SARS-CoV-2 infection on serum hepatocyte growth factor (HGF) concentrations as a marker of the prognosis of coagulation in long COVID-19 survivors. This review discusses the pathophysiology between COVID-19 and HGF, IL-6, and D-dimer. 

## 2. Long COVID-19

Nalbandian et al. (2021) defined post-acute COVID-19 as persistent symptoms and/or long-term or delayed complications of SARS-CoV-2 infection more than 4 weeks from the onset of symptoms. Based on the recent literature, the disease is further divided into two categories: (1) continuing or subacute COVID-19 symptoms, which comprise anomalies and symptoms that started 4–12 weeks after acute COVID-19; and (2) chronic or post-COVID-19 syndromes, which include symptoms and abnormalities that persist or appear more than 12 weeks after the onset of acute COVID-19 and are not associated with an alternative diagnosis [13]. Numerous ongoing medical disorders can be classified as post-COVID-19 conditions. Weeks, months, or even years may pass with this syndrome. According to a longitudinal study, 68% of post-COVID-19 patients at Capital Medical University Beijing who enrolled on 7 January 2020 still showed post-COVID-19 symptoms after six months. Moreover, 55% of people are still feeling the long-term impacts of COVID-19 even after two years [14].

Patients who have been infected with the virus that causes COVID-19 might develop post-COVID-19 conditions, even if they have a minor sickness or no symptoms of COVID-19. Post-COVID-19 conditions are more common in persons who have severe COVID-19 disease. People who have recently contracted COVID-19 but have not received the COVID-19 vaccine may also be at an increased risk of having a post-COVID-19 disease [12]. Clinical symptoms of SARS-CoV-2 infection in patients might range from asymptomatic to life-threatening sickness. Adults infected with SARS-CoV-2 can be classified into the following illness severity levels in general. Clinical guidelines and clinical studies may have different or overlapping criteria for each category, and a patient’s clinical condition may alter over time [15].

1. Asymptomatic or pre-symptomatic infection: Those who test positive for SARS-CoV-2 utilizing virological assays (such as nucleic acid amplification tests [NAAT] or antigen testing) but who do not exhibit symptoms resembling COVID-19.
2. Mild illness: People who experience any of the COVID-19 symptoms (such as fever, cough, sore throat, malaise, headache, muscular aches, nausea, vomiting, diarrhea, and loss of taste and smell) but do not experience dyspnea, shortness of breath, or abnormal chest imaging.
3. Moderate disease: Patients have an oxygen saturation (SpO_2_) of 94% or higher in room air at sea level with clinical or imaging signs of lower respiratory disease.
4. Severe disease: individuals with SpO_2_ levels below 94% in room air at sea level, arterial PaO_2_/FiO_2_ ratios below 300 mm Hg, respiratory rates above 30 breaths per minute, or pulmonary infiltrate levels above 50%.
5. Critical illness: People who have multiple organ malfunctions, septic shock, or respiratory failure [15].

Based on a meta-analysis and article review conducted by Lopez-Leon et al. (2021), there are more than 50 clinical manifestations that are long-term effects of COVID-19 [16]. Fatigue, dyspnea, chest pain, loss of taste or smell, cognitive abnormalities, arthralgia, and a lower quality of life are among the frequently mentioned long-term side effects of the SARS-CoV-2 virus [17]. Such persistent symptoms may be caused by cellular damage, a strong innate immune response with inflammatory cytokine production, and a procoagulant condition brought on by SARS-CoV-2 infection [18,19]. Additionally, a review article reported the symptoms of subacute/sustained and chronic COVID-19 in a number of organ systems, including hematology, the lung, cardiovascular neuropsychiatry, renal, endocrine, gastrointestinal, hepatobiliary, dermatological, and multisystem inflammatory syndrome in children (MIS-C) [13]. Also, a comprehensive review to address the putative pathophysiology underlying the persisting symptoms of long COVID-19 can be seen in Table 1 [20].

A retrospective study by Patel et al. (2020) described post-COVID-19 syndrome in the hematological system. A venous thromboembolism (VTE) of post-acute COVID-19 reached <5%, where in 163 patients there were 2.5% of patients without post-hospital thromboprophylaxis experienced thrombotic events 30 days after discharge, including segmental pulmonary embolism, intracardiac thrombus, thrombosis arteriovenous fistula and ischemic stroke [31].

## 3. Coagulation and Inflammation on Long-Term Post-Viral Effects

SARS-CoV-2, or the acute respiratory syndrome coronavirus, primarily affects the lungs and upper airways. Multiple organs can spread depending on how severe the infection is, and systemic COVID-19 is linked to a high rate of thromboembolic consequences and the possibility of multiorgan failure. Thrombotic inflammation and endothelial vascular damage are two important new elements in the pathogenesis of COVID-19 [32].

COVID-19-associated coagulopathy is consistent with a hyperinflammatory and hypercoagulable state [33]. Release of pro-inflammatory cytokines [34], platelet activation and platelet–leukocyte interactions [35], endothelial injury [36,37], complement activation [38], disruption of regular coagulant pathways [39], hypoxia [40] and neutrophil extracellular traps [41], are some of the mechanisms underlying thromboinflammation. These mechanisms are similar to the pathophysiology of thrombotic microangiopathy syndromes [42]. The severity and duration of a hyper-inflammatory state are likely related to the risk of thrombotic problems in the post-acute COVID-19 phase, but the length of time this lasts is unknown. Hyper-inflammatory and hypercoagulable state COVID-19-associated coagulopathy could account for the abnormally high rates (20–30%) of thrombotic problems in acute COVID-19 as opposed to bleeding complications [38].

The pathophysiology of severe community-acquired pneumonia brought on by other viruses or bacteria is similar to that of ARDS brought on by the severe acute respiratory syndrome coronavirus 2 (SARS-CoV-2). Tumor necrosis factor (TNF), interleukin-6 (IL-6), and interleukin-1 (IL-1) overproduction led to a cytokine storm, eventually causing hyper-inflammatory, which increases the risk of vascular hyperpermeability, multiorgan failure, and finally mortality when cytokine concentrations fall. High does not get lower with time [42]. When the immune system reacts to infection, coagulation pathways are activated, which causes an overproduction of proinflammatory cytokines and multiorgan damage. Although thrombin’s main job is to activate platelets and turn fibrinogen into fibrin, thrombin also has a number of cellular effects and may increase inflammation through receptor-activated proteinase (PAR), which is primarily PAR-1,5. These effects are regulated by negative feedback loops and physiological anticoagulants like antithrombin III, tissue factor pathway inhibitors, and protein C system inhibitors. All three of these regulatory processes may be compromised during inflammation, leading to lower anticoagulant concentrations as a result of both increased consumption and decreased synthesis. As seen in severe COVID-19 pneumonia, where elevated D-dimer concentrations are a poor prognostic factor and disseminated intravascular coagulation is typical in non-survivors, this impaired procoagulant–anticoagulant balance predisposes to the development of micro thrombosis, disseminated intravascular coagulation, and multiorgan failure [19,43]. 

In COVID-19, coagulopathy is characterized by elevated levels of fibrinogen and D-dimer [44]. According to studies, the expression of coagulation factors that may cause VTE may be significantly influenced by the activation of an inflammatory response. It is crucial to determine whether there is a relationship between the patterns of inflammatory dysregulation markers and abnormal coagulation factor levels in different viral pandemics [45]. Hepatocyte growth factor (HGF) is a marker that plays a role in the prevalence of disseminated intravascular coagulation (DIC). Pro-HGF may be overexpressed by stromal cells in infected patients’ tissues following the release of inflammatory cytokines, which are potent inducers of pro-HGF transcription. Pro-HGF is then converted into procoagulant factor HGF through a variety of mechanisms, including induction of tissue factor expression (TF) by monocytes and upregulation of genes like plasminogen activator inhibitor-1 (PAI-1) and cyclooxygenase-2 (COX-2) [46]. A study by Chung et al. (2010) demonstrated in DIC patients; there is a correlation between HGF and plasma levels of IL-6 as the most highly proinflammatory cytokine in SARS-CoV-2 [47].

The cardiovascular system also expresses the ACE2 receptor broadly. As a result, COVID-19 has numerous cardiovascular concerns. Patients who already have cardiovascular disease are more likely to experience severe adverse effects. Furthermore, myocardial injury caused by severe infections has been linked to death through causing myocardial injury. The last possibility is that COVID-19 patients may have thrombotic and coagulation problems, which would lead to a hypercoagulable condition and a higher frequency of thrombotic and thromboembolic events. Any thrombotic event occurs at a rate of about 16% in patients who need to be hospitalized, ranging from 11.5% in non-intensive care unit (ICU) settings to 29.4% in ICU settings [48].

## 4. COVID-19 Inflammation and Blood Coagulation Biomarkers

From various biomarkers there are several choices of biomarkers that can be used in post-COVID-19 inflammatory and coagulation conditions, as follows:

### 4.1. D-Dimer

D-dimer is an epitope formed when plasmin breaks down fibrin crosslinks. Several disorders, including thrombosis, DIC, and inflammation, raise D-dimer levels. By stimulating the release of various inflammatory cytokines from neutrophils and monocytes, D-dimer can aid in the development of inflammation [49]. Numerous viral infections have been said to cause thrombosis and bleeding as side effects. The development of microvascular thrombus in numerous organs might worsen DIC as a result of an excessive reaction to infection [50]. Tang et al. (2020) found coagulopathy in COVID-19 pneumopathy at an advanced stage with elevated levels of D-dimer and fibrinogen. Due to plasmin-associated hyperactive fibrinolysis and the plasminogen/plasmin system’s involvement in COVID-19 illness, highly elevated D-dimer is produced [19]. Concerns about the cohabitation of venous thromboembolism aggravating ventilation–perfusion mismatch have been raised by the discovery of higher d-dimer levels in COVID-19 patients, and numerous investigations have demonstrated the prevalence of pulmonary embolism. Clinicians would be hesitant to prescribe endogenous anticoagulants to everyone, nevertheless, due to the elevated risk of bleeding and discouragement associated with earlier unsuccessful trials of them in sepsis. It is evident that the effects of coagulation activation go beyond clotting and that the interaction between coagulation and inflammation can profoundly affect disease progression and result in unfavorable outcomes beyond the prevention and management of venous thromboembolism [42].

Various studies state an increase in D-dimer with post-COVID-19 conditions. Fazio et al. (2020) stated after complete clinical recovery from COVID-19, restoration of well-being, and normalization of a molecular swab, 20% of patients had significantly increased D-dimer levels following full clinical recovery of COVID-19, restoration of health, and normalization of a molecular swab. These levels gradually decreased after roughly two weeks of preventive enoxaparin medication [51]. As a result, it is possible that continued elevation of D-dimer levels after a patient has recovered clinically will serve as a warning to identify the processes underlying the frequent long-term effects of SARS-CoV-2 infection. Furthermore, another study by Lehmann et al. (2021) reported that after a median of 3 months following COVID-19, a persistent D-dimer increase was seen in 15% of the individuals who had recovered from COVID-19. These individuals had a more severe COVID-19 infection before [52].

Additionally, several participants who received mRNA injections from Moderna and Pfizer showed an increase in D-dimer, suggesting that they were at risk of clotting. The clinical spectrum ranged from no symptoms to severe, necessitating hospitalization, and D-dimer levels were tested the day before immunization and after 5–7 days [53].

D-dimer may, therefore, be thought of as an easy, trustworthy, and affordable test to monitor and follow COVID-19 patients who need to continue taking low molecular weight heparin or other blood thinners after achieving clinical recovery. But, due to the low specificity of the D-dimer test, there has been an extensive search for alternative or additional biomarkers for specific diagnosis of specific clinical coagulation conditions related D-dimer evaluation [54]. 

### 4.2. Interleukin-6 (IL-6)

Interleukin-6, or as we called it, IL-6, is a pleiotropic proinflammatory cytokine that is produced by a variety of cell types, including lymphocytes, monocytes, and fibroblasts. Bronchial epithelial cell-dependent production of IL-6 is induced by SARS-CoV-2 infection. A major finding in COVID-19 patients is cytokine dysregulation. Recent research in severely ill COVID-19 patients reveals that the excessive and uncontrolled release of proinflammatory cytokines, known as “Cytokine storm syndrome”, in the body of an infected patient during infection is directly associated with the deregulation of the immune response to the virus [55]. 

Significant pro-inflammatory properties are exhibited by IL-6, which plays an important role in cytokine storm syndrome, as elevated serum IL-6 levels correlate with respiratory failure and ARDS, which are mediated by two major signaling pathways: cis and trans [56]. In the cis signaling pathway, when IL-6 interacts with membrane-bound IL-6 receptors (IL-6R) and gp130, downstream Janus kinases (JAKs) and also signal transducer and activator of transcription 3 (STAT3) are activated. Activation of this signaling cascade affects the innate immune system (macrophages, neutrophils, and natural killer cells) and acquired immune system (B and T cells), both of which contribute to CRS in a variety of ways. In trans-signaling, circulating IL-6 binds to the soluble IL-6 receptor (sIL-6R) and, in the majority of somatic cell types, forms a complex with the gp130 dimer. Endothelial cells and other cells that do not express mIL-6R become activated as a consequence of the IL-6-sIL-6R-JAK-STAT3 signaling. It substantially worsens the “cytokine storm” through the release of vascular endothelial growth factor (VEGF), monocyte chemoattractant protein-1 (MCP-1), more IL-6 and IL-8, and decreased endothelial E-cadherin expression. VEGF secretion and decreased E-cadherin expression, both of which increase vascular permeability and leakage, play a role in the etiology of pulmonary dysfunction in ARDS [56].

Enhanced blood levels of IL-6, C-reactive protein (CRP), D-dimer, and ferritin are indicators of increased cytokine release and may be linked to systemic inflammation and hypoxemic respiratory failure caused by COVID-19. The length and/or severity of COVID-19 may be lessened by modifying IL-6 levels or IL-6’s effects [57].

IL-6 levels are markedly increased and linked to poor clinical outcomes in COVID-19 patients. High-quality trials of intervention in this area are urgently needed. Inhibition of IL-6 may be a novel target for therapies for the control of dysregulated host responses in patients with COVID-19 [58]. Evidence showing a direct correlation between circulating IL-6 levels and the severity of COVID-19 infection was reported. In order to control inflammatory and immune reactions, cytokines are essential. Due to its pleiotropic effects, IL-6 stands out as being particularly significant among them. On the other hand, prior research has shown that individuals with respiratory dysfunction have elevated IL-6 levels [59,60], suggesting a possible common mechanism of cytokine-mediated lung damage brought on by COVID-9 infection. The highly pathogenic SARS-CoV-2 also appears to be linked to rapid virus replication and a propensity to infect the lower respiratory tract, leading to an increased response of IL-6-induced acute respiratory distress. As a result, their findings imply that repeated measurements of circulating IL-6 levels may be crucial for detecting illness development in COVID-19-infected individuals [59]. 

### 4.3. Hepatocyte Growth Factor (HGF) 

Hepatocyte Growth Factor (HGF) is produced by mesenchymal cells and functions paracrine in epithelial and endothelial cells as a mitogen, motogen, and morphogen. Tyrosine kinase activation is brought on by HGF’s binding to the c-Met receptors on epithelial cells. Blood coagulation serine proteases, such as tissue-type plasminogen activator (tPA), urokinase, factor XIIa, and factor X, cleave the inactive single-chain precursor (pro-HGF) between Arg and Val to transform it into active HGF. The HGF transforming potency of factor XIIa is 1000 times larger than that of tPA or urokinase, making it the most effective activator of them all. Because thrombin transforms the zymogen of the HGF activator into the active form, this conversion appears to be connected to blood coagulation. Urokinase-type (uPA) and tissue-type (tPA) plasminogen activators, coagulation factor XIIa (the most potent activator), and a serine protease homologous to coagulation factor XII, known as an activator of HGF, which is a substrate of thrombin, are the four known plasminogen family proteases that activate pro-HGF. In addition to encouraging the release of active HGF from granulocytes into the bloodstream, coagulation factor Xa also cleaves HGF in the chain, producing an N-terminal fragment with diminished biological activity but the ability to still bind receptors [36]. Therefore, we can draw a conclusion that an activated blood clotting system raises blood levels of active HGF through a variety of its effectors.

Circulating pro-HGF is in equilibrium with that embedded in the dense tissue extracellular matrix. Here, pro-HGF is mostly secreted by stromal cells and stored by coupling with heparan sulfate in the proximity of target cells, which include endothelial and most epithelial cell types. Vascular damage leads to exposure of pro-HGF-bound matrix to blood coagulation enzymes, HGF formation, and stimulation of MET-expressing cells. Interestingly, HGF is not only a downstream effector of blood coagulation but also an activator, as it increases the transcription of genes controlling hemostasis, such as plasminogen activator inhibitors (PAI-1) and cyclooxygenase 2 (COX-2). PAI-1 on fibrinolysis, while COX-2 catalyzes steps in the synthesis of prostaglandins and prostacyclin, which control platelet function [46]. 

The study by Mosevoll et al., 2015, which examined the numerous soluble inflammatory-related mediators in plasma samples and examined significantly different plasma biomarker profiles between patients with DVT, patients treated for suspected thrombosis but without DVT, and healthy individuals, discussed the role of HGF in coagulation. HGF was one of the mediators the study found to be significantly different between DVT patients and those without thrombus [54]. Another previous study by Perreau et al. (2021) regarding HGF in COVID-19 provides insight into the early pathophysiological events associated with severe COVID-19. Elevated serum HGF concentrations early in symptomatic infection and their association with ICU admission are possible indicators of an ongoing severe respiratory syndrome associated with interstitial pneumonia. HGF upregulation is a physiological counter-regulatory immune response of the host to reduce inflammation, limit lung tissue injury, and promote tissue repair. Consistent with this view, more than 90% of non-ICU patients with moderate respiratory syndrome have low HGF levels, thus identifying HGF as one of the critical pathogenic biomarkers for disease severity and the best predictor of the risk of ICU admission and death in COVID-19 sufferers. Nonetheless, the role of HGF as a biomarker for coagulation in survivors of COVID-19 is unknown [61]. A study by Tamayo-Velasco et al. (2021) explains HGF as a biomarker which substantially related to severe/critical COVID-19 patients at hospital admission and is hence an excellent indicator of poor prognosis and also used as a mortality biomarker [62]. 

When inflammatory cytokines, such as IL-1, which are potent inducers of pro-HGF transcription, are released in infected patients, stromal cells in tissues may overexpress pro-HGF. HGF and plasma IL-6 levels were shown to correlate in DIC patients [47]. It is interesting to note that the association held true for cancer patients as well as for everyone else who is currently dealing with open DIC. It would be intriguing to look into whether HGF levels in the cancer group were associated with DIC scores, at least in those patients who had high levels of inflammatory cytokines [36]. The absence of a connection between HGF level and DIC severity across the entire cancer cohort may be a result of the complex interplay between blood clotting, tumor tissue, and HGF. Tumor cells can actually: (a) produce HGF; (b) secrete PAI-1, which prevents plasminogen activator from cleaving HGF into its component proteins; (c) interfere with blood coagulation in a number of ways, including tissue factor expression and release via microvesicles; production of molecules (like mucin) that control platelet aggregation; and Secretion of cytokines that activate the endothelium. Levels of pro-HGF and active HGF are probably unrelated to coagulopathy’s supportive mechanisms in this complicated situation [46]. Additionally, it was revealed in a study by Chung et al. (2010) that HGF influences blood clotting and promotes the growth of DIC [47]. 

HGF, also known as the scatter factor, plays a crucial role in various biological processes, including the blood clotting system. While HGF is not directly involved in blood clotting itself, it influences several factors and pathways that regulate the process. It primarily acts on hepatocytes, endothelial cells, and platelets to modulate their functions and interactions, which indirectly affects blood clotting [63,64].

#### 4.3.1. Endothelial Cell Function

HGF promotes endothelial cell proliferation and migration, leading to the formation of new blood vessels. This angiogenic effect is essential during wound healing and tissue repair, where the development of new blood vessels is crucial for delivering oxygen and nutrients. Proper vascularization is essential for the blood clotting process, as it ensures an adequate blood supply to the injured area [46]. According to reports, COVID-19 may be impacted by endothelial dysfunction and a loss of endothelial barrier function. Heparanase, an endothelial glycocalyx-degrading enzyme, is widely known to play a role in vascular leakage and inflammation. Heparanase is inhibited by low molecular weight (LMW) heparins [65]. Heparinases are enzymes that selectively split the chains of heparin and heparan sulfate by cleaving the glycosidic bond forming between hexosamines and uronic acids, producing disaccharide and oligosaccharide products. Heparin is well known as an anti-coagulant medication but is also implicated in biological processes, e.g., inflammation, cancer, angiogenesis, and viral and bacterial infections. Heparin and heparan sulfate are gaining attention due to their potential as therapeutics and may play a role in the COVID-19 infection, according to recent research. LMW heparin, which has taken the role of heparin in various clinical uses, is produced by the industrial exploitation of heparinases’ capacity to cleave heparin chains selectively. Heparinases are also used to analyze the structural properties of heparin and heparan sulfate, neutralize heparin in blood, and remove heparin’s inhibitory effects on other enzymes [66].

#### 4.3.2. Platelet Activation and Aggregation

HGF stimulates platelet activation, resulting in the release of various factors from platelet granules. These factors, such as ADP, thromboxane A2, and serotonin, contribute to platelet aggregation and the formation of a stable blood clot. HGF also enhances platelet adhesion to the endothelial cells lining blood vessels, which is a critical step in initiating clot formation [63].

#### 4.3.3. Hepatocyte Production of Coagulation Factors

The liver is the only primary site of production for many coagulation factors, including Factor Xa. HGF stimulates the growth and regeneration of hepatocytes, which ensures an adequate supply of coagulation factors for the blood clotting cascade. Disruptions in HGF signaling can potentially impact the synthesis and availability of these factors, leading to deficiencies and impairments in the blood clotting system [67].

Recent failures of clinical trials targeting Factor Xa inhibitors have highlighted the need for alternative drug targets or advancements. Factor Xa inhibitors are anticoagulant medications that work by blocking the activity of Factor Xa, a key enzyme involved in the blood clotting cascade. While these inhibitors have shown efficacy in preventing and treating thrombotic disorders, some patients experienced adverse events, including bleeding complications. Identifying alternative drug targets or inhibitors is crucial for advancing anticoagulant therapies. Potential strategies could involve targeting other enzymes involved in the coagulation cascade, such as thrombin or Factor VIIa, or developing novel approaches to modulate platelet activation and aggregation. Additionally, advances in understanding the signaling pathways and interactions involving HGF could offer new insights for therapeutic interventions in the blood clotting system [68,69]. Also, further research is needed to explore alternative drug targets and develop safer and more effective anticoagulant therapies, ensuring a balance between preventing thrombosis and minimizing the risk of bleeding complications. 

**Figure 1:**
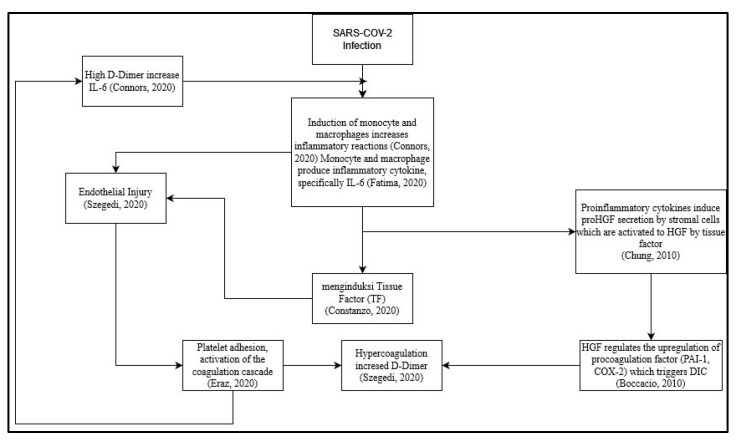
Proposed schematic for correlation between hepatocyte growth factor (HGF) with D-dimer and Interleukin 6 (IL-6) as a prognostic marker of coagulation and inflammation on long-term effects of COVID-19 survivor [37,46,47,56,70,71,72].

However, until now, the role of HGF in coagulation risk in post-COVID-19 patients is unknown, and how it correlates with D-dimer as a coagulation factor and interleukin 6 as a proinflammatory cytokine that can induce coagulation, especially due to long-term cytokine storms. Here, we try to make a suggested scheme based on our review before (Figure 1). One of the proposed mechanisms is the direct viral infection of endothelial cells that line blood vessels. This infection can cause endothelial dysfunction, leading to the release of procoagulant factors and impaired regulation of anticoagulant mechanisms. Additionally, the dysregulated immune response and cytokine storm can further promote a prothrombotic state [70]. Other further studies are needed to assess the correlation between each criterion and alteration or dysregulation of the immune system.

In the proposed schematic (Figure 1), infection from SARS-CoV-2 can trigger an inflammatory process in the body [37]. The inflammatory process during SARS-CoV-2 infection is induced by monocytes and macrophages [56]. The induction of monocytes and macrophages triggers the emergence of proinflammatory cytokines, especially IL-6 [56]. Proinflammatory cytokines induce proHGF secretion by stromal cells, which are then activated by Tissue Factor to become HGF [47]. HGF regulates the upregulation of pro-coagulation factors (PAI-1&COX-2), which trigger disseminated intravascular coagulation (DIC) [46]. IL-6 cytokines induce Tissue Factor [71] and cause endothelial injury [72]. The occurrence of DIC causes hypercoagulation and increases the concentration of D-dimer [37]. The occurrence of endothelial injury causes platelet adhesion, thereby activating the coagulation cascade [73]. Hypercoagulation causes high D-dimer concentrations, thereby increasing IL-6 concentrations [71].

## 5. Conclusions

This review article describes studies that correlate the long-term effects of COVID-19, especially in inflammatory conditions followed by coagulation with widely used biomarkers such as IL-6 for inflammation and D-dimer for coagulation. The coagulation marker that is routinely used today is D-dimer, while the inflammatory marker that is commonly and routinely used today is IL-6. However, the correlation of HGF biomarkers with coagulation and inflammation in COVID-19 survivors is still unknown, so further studies are needed to clarify the correlation between HGF and coagulation and inflammation markers that have been used routinely so that it can provide information for COVID-19 survivors about the risk of the condition. DIC may lead to certain conditions, one of which is stroke or other cardiovascular diseases.

## Figures and Tables

**Table 1:** Review of the body system affected by long COVID-19 (study, symptoms, and mechanism).

Systems	Study	Symptoms	Mechanism	Refs.
Cardiovascular system	A study cohort of German patients who recently recovered from COVID-19 infection: CMR (*cardiovascular magnetic resonance*) revealed cardiac involvement in 78 patients (78%) and ongoing myocardial inflammation in 60 patients (60%). A prospective cohort study included patients who had been hospitalized with COVID-19; in total, 1306 patients proved the illness trajectory of COVID-19 includes persisting cardio-renal inflammation, lung involvement, hemostatic pathway activation, and impairments in physical and psychological function	Fatigue Dyspnea Chest pain	Persistent vascular inflammation Macrovascular vascular inflammation Microvascular inflammation: increased level of cytokines, circulating endothelial cells, coagulation activation, microvascular retinal impairment (at autopsy, evidence of endothelial cells and cardiomyocytes viral invasion with signs of structural alterations) Auto-immunity: auto-antibodies able to modulate the cardiac frequency and vascular tone (acting as receptor agonists on the b2-adrenoceptor, the a1-adrenoceptor, angiotensin II AT1-receptor, angiotensin, and endothelin receptors) Persistent alteration of coagulation (a sustained increase of D-dimer levels)	[21,22]
Neurology	A Study case from a 56-year-old man from Italy who was previously healthy and went to the hospital after experiencing fever and dyspnea for five days was found to have long-term brain disorders in post-COVID-19 neurological syndrome (PCNS) A total of 51 patients with two subtypes of COVID-19 (19 mild and 32 severe) and no obvious lesions on the standard MRI. After 3 months, the discharged patient showed that the inflammatory storm’s indirect brain damage may affect cerebral volume, CBF, and WM tracts. The hypoxemia brought on by COVID-19 and the malfunction of the vascular endothelium may possibly be a factor in the neurological alterations.	Cognitive and mental health disorders Pain Headache Fatigue Anosmia/ageusia Neuropathy	Functional brain disturbances Hypometabolic activity in various cerebral zones Reduced activity of the GABA inhibition Neuroinflammation and brain microstructural modifications Micro-structural, volumetric, and vascularization disorders	[23,24]
Respiratory	Some patients still exhibit persistent CT abnormalities six months after the acute infection, including the disappearance of (ground-glass opacity) GGOs that were present in the early stages of recovery as well as the development or maintenance of changes that are indicative of fibrosis, such as reticulation with or without parenchymal distortion. A total of 351 patients with 2 subtypes of COVID-19 (19 mild and 32 severe) with no specific neurological manifestations at the acute stage and no obvious lesions on the conventional MRI 3 months after discharge demonstrated that indirect injury related to the inflammatory storm may cause brain damage, altering cerebral volume, CBF, and WM tracts.	Dyspnea Chest pain Cough	Persistent inflammation and dysregulated host response of lung repair Increased plasma biomarkers of lung inflammation and fibrosis (Lipocalin 2, Matrix metalloproteinase-7, Hepatocyte growth factor) Persisting inflammation in the lungs, mediastinal lymph nodes, spleen, and liver Involvement of iron homeostasis disturbances in end-organ damage Relationship between metabolic abnormalities and lung sequelae	[25,26]
Gastro-intestinal system	Case study of a 71-year-old patient, two episodes of frank blood in stools in the last 24 h manifested with features resultant of both pulmonary thromboembolism and volume depletion secondary to gastrointestinal blood loss. From October to December 2020, 139 post-COVID-19 patients were included. These patients had acute COVID-19 and were one to eight months out. Significant differences in the microbiota diversity between post-COVID-19 patients and controls show gut dysbiosis even months after the acute disease has resolved.	No specific symptom	Gut microbiota modifications after recovery Decreases gut commensals with known immunomodulatory potential Perturbed composition of microbiota correlated inflammation biomarkers	[27,28]
Immune system	Case records of the 18 children with a predominant presentation of epileptics, Guillain–Barré syndrome, stroke, demyelinating syndromes, and autoimmune encephalitis. A total of 70 cohort participants that represented a wide range of initial COVID-19 disease presentations, from those with no or mild symptoms to those requiring hospitalization or treatment in an intensive care unit (ICU), showing important patterns across assays measuring adaptive and humoral immune responses for various clinical factors such as initial clinical severity defined by hospitalization or ICU care, pre-existing pulmonary disease, and PASC.	Multi-system symptoms	Persistent immune inflammatory response impairing organ functioning Remaining inflammation in blood samples analysis, long-lasting phenotypic and functional disorders of lymphocytes, decreased amounts of dendritic cells, and persisting alterations of activation markers Signs of mild organ impairment at magnetic resonance imaging and FDG PET/CT Autoimmunity: auto-antibodies against the nociceptive receptors, immunomodulatory proteins (including cytokines, chemokines, complement components, and cell-surface proteins), and tissue components Persistence of the SARS-CoV-2 nucleic acids in tissues Multisystem Inflammatory syndrome in children (MIS-C)	[29,30]

## Data Availability

No new data were created or analyzed in this study. Data sharing is not applicable to this article.
